# Personalized Assessment of Mortality Risk and Hospital Stay Duration in Hospitalized Patients with COVID-19 Treated with Remdesivir: A Machine Learning Approach

**DOI:** 10.3390/jcm13071837

**Published:** 2024-03-22

**Authors:** Antonio Ramón, Andrés Bas, Santiago Herrero, Pilar Blasco, Miguel Suárez, Jorge Mateo

**Affiliations:** 1 Department of Pharmacy, University General Hospital, 46014 Valencia, Spain; ramon_ant@gva.es (A.R.); bas_and@gva.es (A.B.); herrero_sangon@gva.es (S.H.); blasco_pil@gva.es (P.B.); 2 Medical Analysis Expert Group, Institute of Technology, University of Castilla-La Mancha, 16002 Cuenca, Spain; 3 Department of Gastroenterology, Virgen de la Luz Hospital, 16002 Cuenca, Spain; 4 Medical Analysis Expert Group, Instituto de Investigación Sanitaria de Castilla-La Mancha (IDISCAM), 45071 Toledo, Spain

**Keywords:** COVID-19, hospital stay, machine learning, mortality, SARS-CoV-2, remdesivir, XGB

## Abstract

**Background:** Despite advancements in vaccination, early treatments, and understanding of SARS-CoV-2, its impact remains significant worldwide. Many patients require intensive care due to severe COVID-19. Remdesivir, a key treatment option among viral RNA polymerase inhibitors, lacks comprehensive studies on factors associated with its effectiveness. **Methods:** We conducted a retrospective study in 2022, analyzing data from 252 hospitalized COVID-19 patients treated with remdesivir. Six machine learning algorithms were compared to predict factors influencing remdesivir’s clinical benefits regarding mortality and hospital stay. **Results:** The extreme gradient boost (XGB) method showed the highest accuracy for both mortality (95.45%) and hospital stay (94.24%). Factors associated with worse outcomes in terms of mortality included limitations in life support, ventilatory support needs, lymphopenia, low albumin and hemoglobin levels, flu and/or coinfection, and cough. For hospital stay, factors included vaccine doses, lung density, pulmonary radiological status, comorbidities, oxygen therapy, troponin, lactate dehydrogenase levels, and asthenia. **Conclusions:** These findings underscore XGB’s effectiveness in accurately categorizing COVID-19 patients undergoing remdesivir treatment.

## 1. Introduction

The Coronavirus Disease 2019 (COVID-19) pandemic began in late December 2019 in Wuhan, China, and was caused by a new beta-coronavirus called Severe Acute Respiratory Syndrome Coronavirus 2 (SARS-CoV-2) [1]. According to the World Health Organization (WHO), as of 13 December 2023, the pandemic has led to nearly 7 million deaths worldwide [2]. The Omicron variant emerged at the end of 2021, replacing the Delta variant. This variant appears to be less clinically severe than earlier ones, likely due to widespread vaccination [3]. Additionally, various treatments have helped reduce hospitalization and mortality rates [4,5].

COVID-19 is a highly contagious disease that poses significant risks of severe illness and death. It can result in bilateral pneumonia, severe respiratory failure requiring invasive mechanical ventilation (IMV), and damage to multiple organs, sometimes resulting in tragic outcomes [6].

SARS-CoV-2 affects various organs, as observed in autopsy findings, including the kidneys, heart, intestines, liver, and brain. However, it primarily targets the respiratory system [7]. Most infected individuals will have symptoms like cough, fever, fatigue, and muscle pain. However, a small percentage may develop severe inflammation in the lungs, leading to acute respiratory distress syndrome (ARDS) [8]. Additionally, it can cause complications such as kidney problems, coagulation disorders, or shock, resulting in mortality rates of over 30% [9].

The virus enters cells using structures like angiotensin-converting enzyme 2 and transmembrane serine protease 2, which are present in the respiratory tract, cornea, and gastrointestinal cells [10].

In COVID-19, factors contributing to the severity and progression of the infection include advanced age, various health conditions, and changes in lab results. Elevated levels of certain markers like C-reactive protein (CRP); lactate dehydrogenase (LDH); ferritin; procalcitonin; and proinflammatory cytokines such as interleukin (IL)-6, IL-2, IL-1β, TNF-α, and G-CSF have been identified as significant indicators [11]. Moreover, in severe COVID-19 cases, the ratio of neutrophils to lymphocytes is often high [12].

Currently, widespread vaccination is the most effective public health measure in the ongoing fight against SARS-CoV-2. While only a handful of drugs are effective in treating severe cases of COVID-19, early diagnosis and prompt treatment initiation, along with nutritional and organ support, can significantly improve outcomes.

Remdesivir is a bioactive molecule that has demonstrated in vitro activity against SARS-CoV-2, along with prophylactic and therapeutic efficacy in non-clinical models of other viruses, such as SARS-CoV, MERS-CoV, and Ebola [12]. In October 2020, it became the inaugural drug authorized by the Food and Drug Administration (FDA) for the treatment of COVID-19. Based on various clinical trials, its use was approved for adults and pediatric patients aged 12 and older, weighing at least 40 kg, for the treatment of COVID-19 requiring hospitalization [13,14,15].

The preliminary findings from the WHO-funded Solidarity trial revealed that lopinavir, hydroxychloroquine, interferon (IFN)-β1a, and remdesivir had little to no effect on hospitalized COVID-19 patients, as indicated by overall mortality, initiation of ventilation, and hospital stay duration [16]. The final results highlight the ineffectiveness of the drugs under investigation, except for remdesivir. While it does not significantly impact COVID-19 patients already on ventilation, it does show a modest effect on mortality or progression to ventilation (or both) in other hospitalized patients [17].

Currently, little is known about the predictive factors linked to poor outcomes in hospitalized COVID-19 patients treated with antivirals in general and specifically with remdesivir in clinical practice.

The goal of our study is to use machine learning (ML) models to categorize hospitalized COVID-19 patients undergoing remdesivir treatment based on the risk of mortality and/or hospital stay duration. ML, a subset of artificial intelligence (AI), employs statistical and mathematical algorithms to extract patterns from the data, aiding in making complex decisions [18]. Unlike classical statistical models created for inferences about variable relationships, these models are created to accurately predict outcomes using data from various factors.

AI tools have been implemented in various areas to combat COVID-19, including drug and vaccine discovery or repurposing [19,20].

To our knowledge, this is the first study to develop, compare, and validate six supervised ML models predicting factors associated with a high risk of mortality and/or hospital stay duration in patients with SARS-CoV-2 infection undergoing treatment with remdesivir.

## 2. Materials and Methods

### 2.1. Data Source

Patient information was systematically gathered from various internal hospital channels, utilizing two primary sources: (1) the electronic medical records (EMR) system, equipped with specialized modules for documenting clinical analysis results, radiological imaging findings, and electronic medical prescriptions, and (2) the intensive care unit (ICU) electronic prescription program. This comprehensive approach facilitated the synthesis of extensive data, enabling the methodical development of a personalized data collection questionnaire (DCQ) for each individual patient.

### 2.2. Study Design and Population

A retrospective observational study was conducted at a high-complexity tertiary hospital. Initially, 285 patients began remdesivir treatment, but 33 were excluded for not reaching the minimum required dose. Exclusions were based on various criteria, including a glomerular filtration rate < 30 mL/min, mortality or hospital discharge, clinical decision, symptom onset beyond 7 days of starting remdesivir, and a negative diagnostic test. Ultimately, the study focused on 252 patients (58.3% male) admitted with microbiologically confirmed SARS-CoV-2 between 1 January and 31 December 2022, using reverse transcription-polymerase chain reaction (RT-PCR) from nasopharyngeal swabs.

In this study, inclusion criteria involved patients ≥ 12 years old and weighing ≥ 40 kg, admitted to the hospital with a COVID-19 diagnosis. Selected patients met specific requirements for remdesivir administration according to the hospital’s internal protocol at the study’s outset. These criteria included symptom onset ≤ 7 days before the first remdesivir dose, no need for oxygen therapy or low-flow oxygen therapy, and meeting at least two of the following three conditions: (1) respiratory rate (RR) ≥ 24 breaths per minute; (2) baseline oxygen saturation (SpO_2_) < 94% in ambient air; and (3) PAFI index (PaO_2_/FIO_2_) < 300 mmHg. Patients were required to have received a minimum of three doses of the drug. The approved remdesivir regimen consisted of an initial 200 mg loading dose administered intravenously over 30–120 min in 100–250 mL of sterile, pyrogen-free 0.9% sodium chloride solution, followed by maintenance doses of 100 mg for a duration ranging from 5 to 10 days, depending on the patient’s level of immunosuppression. Participants agreed to take part in the study after being informed about it, and the study was approved by the Ethics Committee of the General University Hospital of Valencia.

### 2.3. Study Data

The DCQ collected information on demographic, clinical, and laboratory data, organized into 8 sections:

1. Patient profile: This section covered demographic factors like age and sex, as well as clinical aspects including:
  (a) Weight and height or body mass index.
  (b) Presence of comorbidities, such as smoking, obesity, hypertension, diabetes mellitus (DM), chronic obstructive pulmonary disease (COPD), asthma, and other chronic respiratory conditions. Details on other serious underlying conditions are provided in an open-text section. The number of comorbidities was categorized (1, 2, 3, and >3).
  (c) Pre-admission pharmacological treatment: This included the use of angiotensin-converting enzyme inhibitors/angiotensin receptor blockers, non-steroidal anti-inflammatory drugs, antihistamines, and/or montelukast, as well as information on the type of COVID-19 vaccine received and the number of doses administered.
  (d) COVID-19 Symptoms: A detailed list of symptoms was recorded, including fever, cough, dyspnea, fatigue, loss of taste or smell, headache, myalgia, sore throat, nasal congestion, rhinorrhea, conjunctivitis, rash, nausea, vomiting, and diarrhea.
2. Initial hospitalization details:
  - Date of admission to the emergency department.
  - Date of admission to the hospital.
  - Date of symptom onset.
  - Date of microbiological confirmation of SARS-CoV-2 infection.
  - Any life support limitations and date implemented.
  - Whether the patient needed ICU admission.
3. ICU admission details:
  - ICU admission date.
  - Mortality risk assessed by the CURB-65 scale.
  - Level of consciousness evaluated using the Glasgow Coma Scale.
  - Other clinical variables in the first 24 h: fever (≥38 °C), RR > 24 breaths/minute, systolic blood pressure < 90 mmHg, SpO_2_, and number of lung quadrants affected in imaging tests (ranging from 1 to 4).
  - Severity of illness assessed using the APACHE II scale within the first 24 h of admission
  - Patient’s condition evaluated using the SOFA scale during their ICU stay.
4. Analytical and radiological data overview:

This section covers laboratory tests completed just after hospital admission (in the emergency department or upon admission) before starting remdesivir treatment, as well as those performed after completing the remdesivir treatment.

The included laboratory parameters were the PAFI index; FIO_2_; SpO_2_; leukocytes; neutrophils; lymphocytes; monocytes: platelets; mean corpuscular volume; hemoglobin; erythrocyte sedimentation rate; CRP; aspartate aminotransferase; alanine aminotransferase; LDH; blood urea nitrogen; serum creatinine; albumin and/or total proteins; total cholesterol; procalcitonin; lactic acid, bicarbonate, and pH; creatine phosphokinase and/or troponin; and D-dimer and ferritin.

Additionally, the determination of cycle threshold (Ct) values from the virus RT-PCR at the start of treatment (or the closest available) is included. Ct is a semi-quantitative value inversely related to the amount of RNA in the sample. The SARS-CoV-2 antigenic variant is also identified.

A classification of pulmonary radiological status was included:

- Affected side (bilateral or unilateral);
- Type of lung injury (ground-glass opacity, consolidation, or mixed);
- Density pattern (patchy, confluent, or mixed).

5. Pharmacological treatment during hospitalization:

The medications considered included drugs that modulate inflammation and the immune system, such as IL-6 receptor antagonists (e.g., tocilizumab and sarilumab), IL-1 receptor antagonist (anakinra), Janus kinase inhibitors (e.g., baricitinib and tofacitinib), or Bruton’s tyrosine kinase inhibitors (e.g., ibrutinib and acalabrutinib). Additionally, other medications like immunosuppressants (e.g., corticosteroids, cyclosporine, and tacrolimus) or immunoglobulins were considered. Antibiotics, vasopressors, and low-molecular-weight heparin at prophylactic doses were also part of the treatment plan. For each medication, the dosage and duration of treatment were recorded.

The patient’s initial condition upon starting remdesivir therapy was categorized into two groups: (a) patients not needing extra oxygen and (b) patients requiring low-flow oxygen. The patient’s clinical status on days 7 and 14 after the first dose of remdesivir was documented as follows: (a) discharged from the hospital and resumed normal activities; (b) discharged from the hospital but with difficulties resuming normal activities; (c) hospitalized without needing extra oxygen; (d) hospitalized needing extra oxygen but not IMV; (e) hospitalized needing IMV; and (f) deceased.

6. Microbiological testing:

We considered the isolated microorganism in every case. Tests included tracheal aspirates, blood cultures, detection of influenza and/or coinfection, as well as tests for pneumococcal and legionella antigens in urine.

7. Medical procedures during hospitalization:

The included procedures were as follows:

- Hemodialysis/hemofiltration;
- Oxygen therapy;
- Non-invasive ventilation (NIV);
- IMV;
- Extracorporeal membrane oxygenation;
- Prone ventilation.

8. Patients’ final outcomes:

Complications during hospitalization were noted, including ARDS, sepsis, septic shock, nosocomial pneumonia (non-COVID-19), other nosocomial infections (non-COVID-19, non-pneumonia), acute renal failure, and acute hepatic failure.

The clinical benefit consisted of symptom improvement (fever, cough, etc.), along with improved radiological findings and/or a PAFI index ≥ 300 mmHg or SpO_2_ > 93 without oxygen support within the first 5, 14, or 28 days, depending on the length of hospitalization.

Time taken to clear SARS-CoV-2 was considered, and outcomes were divided into hospital discharge or mortality. Collected data included length of hospital stay and date of ICU discharge. Any rehospitalizations within 7 days post-discharge were also documented.

To evaluate remdesivir’s effectiveness, information on overall in-hospital mortality and duration of hospitalization was recorded. Length of hospital stay was defined as the period from admission to death or discharge.

### 2.4. Method

#### 2.4.1. Model Development

In this study, the extreme gradient boost (XGB) method was used as the reference algorithm, thanks to its notable features, such as fast execution, scalability, and high processing capability through parallel computing [21,22]. XGB consistently outperforms other algorithms in accurately solving various data science problems [22,23,24]. Additionally, a comparative analysis was conducted with other supervised ML systems.

Considering a dataset S = *x_j_*, *y_j_*, the XGB model was formulated using the following equation:

(1) $$\hat{y_{j}}=\sum_{p=1}^{P}t_{p}\left(x_{j}\right)$$

where *x_j_* stands for the input vector with *m* time variables, $\hat{y_{j}}$ denotes the predicted output, *y_j_* shows the output, *t_p_* represents a tree with leaf weight *w_p_* and structure *u_p_*, *j* = 1; 2; …; *n*, and *P* is the total number of trees.

The formulated objective function for the proposed method is expressed in Equation (2). Employing a second-order Taylor expansion is integral to improving prediction accuracy in approximating the XGB objective function [21].

(2) $$R=\sum_{j}r\left(\hat{y_{j}},y_{j}\right)+\sum_{p}\Psi \left(t_{p}\right)$$

In Equation (3), *fp* stands for the number of leaves on the tree. The function *R*() penalizes method complexity. The learning rate is represented by λ, and *wp* is the leaf score vector. To control system complexity weight, a parameter *γ* is used. The goal is to optimize Equation (2) [22].

(3) $$\Psi \left(t_{p}\right)=\lambda f_{p}+\frac{1}{2}\gamma {\left\|\omega_{p}\right\|}^{2}$$

In this study, we tested several ML algorithms to evaluate the performance of our proposed method. We chose the top-performing five algorithms from those widely recognized in the scientific community. These include decision trees (DT) [25], Gaussian naive Bayes (GNB) [26], Bayesian linear discriminant analysis (BLDA) [27], K-nearest neighbors (KNN) [28], and support vector machines (SVM) [29]. We built the models using the MatLab Statistical and Machine Learning Toolbox (MatLab 2022a; The MathWorks, Natick, MA, USA). The dataset was split into two parts, with 70% used for training and the remaining 30% for testing, ensuring that patient information was not shared between the sets. To validate the results and prevent overfitting, we conducted 5-fold cross-validation.

Optimizing the ML algorithms involves adjusting various hyperparameters during the training phase. Bayesian techniques were employed in this study to determine optimal hyperparameter values. This optimization method significantly improves the outcomes of the developed methods.

**Figure 1:**
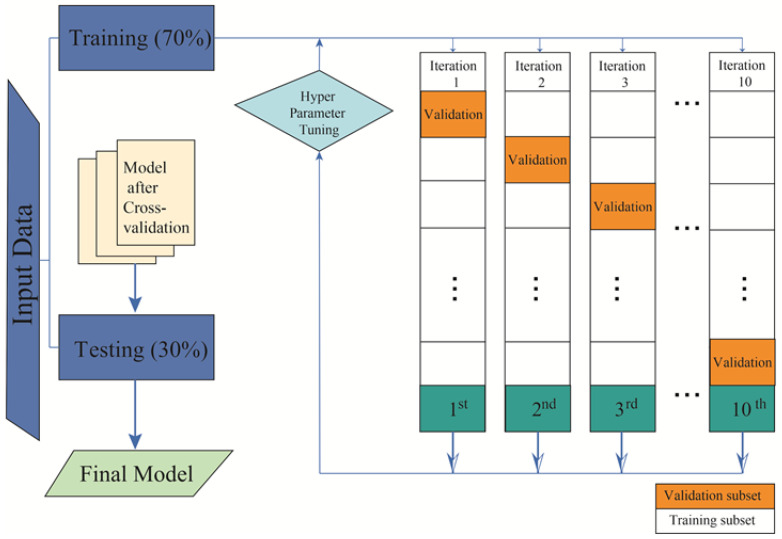
This figure illustrates the framework employed in the training and testing processes of this study.

Throughout all simulations, 100 iterations were executed to derive mean and standard deviation values in a uniformly random manner. This systematic approach mitigates the impact of noise, facilitating the calculation of relevant values and ensuring the attainment of statistically valid results [30]. The procedural phases employed in this study are delineated in Figure 1. Initially, subjects for study were selected, followed by the implementation of the database and subsequent training and validation of ML methods.

#### 2.4.2. Performance Evaluation

In this study, various methods were compared using the following metrics: specificity, precision (positive predictive value), recall (sensitivity), balanced accuracy, degenerate Youden index (DYI), *F*_1_ score, Matthew’s correlation coefficient (MCC), Cohen’s Kappa index (CKI), receiver operating characteristic (ROC), and area under the curve (AUC) [30]. The *F*_1_ score is defined as

(4) $$F_{1}\;score=2\frac{Precision\cdot Recall}{Precision+Recall}$$

MCC was additionally employed to evaluate the performance of the ML methods, and it is defined as

(5) $$MCC=\frac{TP\cdot TN-FP\cdot FN}{\sqrt{\left(TP+FP\right)\left(TP+FN\right)\left(TN+FP\right)\left(TN+FN\right)}}$$

where TP denotes the number of true positives, TN represents the number of true negatives, FP is the number of false positives, and FN corresponds to the number of false negatives. CKI was used to assess the overall performance of the system [31].

## 3. Results

In this section, we discuss the results obtained from patient records used for training and validation to identify predictors of increased in-hospital mortality and hospital stay in COVID-19 patients treated with remdesivir. We compare the performance of our proposed system with various supervised ML classification methods widely accepted in the scientific community.

Table 1 presents the performance outcomes regarding the mortality associated with various classification methods, including DT, BLDA, GNB, KNN, SVM, and our novel XGB system. It is noteworthy that GNB and BLDA-based approaches demonstrate a relatively lower balanced accuracy, falling short of the 82% benchmark. Conversely, DT and SVM techniques exhibit superior classification prowess, nearing a balanced accuracy of 90%, surpassing the effectiveness of GNB and BLDA. In contrast, the KNN method achieves a result that is closest to the proposed XGB method, resulting in improved predictive capabilities. Particularly, the XGB system achieves an outstanding score surpassing 95%, showcasing remarkable performance in classification tasks.

Table 2 presents the performance results for the hospital stay variable using the same classification methods. As can be observed, the outcomes are similar to those of the mortality variable, with XGB achieving a balanced accuracy exceeding 94%.

KNN and DT stand out as the algorithms that come closest to XGB in terms of precision and recall values, surpassing SVM and notably outperforming BLDA and GNB in results. Furthermore, this trend is evident in Table 1 and Table 2, particularly regarding the *F*_1_ score parameter, where XGB demonstrates notably high values, indicating enhanced classification performance.

To evaluate how well the suggested XGB system categorizes COVID-19 patients treated with remdesivir, identifying those with a higher risk of mortality and/or extended hospital stay, we computed several common parameters from the literature. These include AUC, MCC, DYI, and the kappa index. MCC stands out as particularly reliable, as it provides a high score only when predictions are accurate across all four categories of the confusion matrix.

The outcomes of these four categories (true positives, true negatives, false positives, and false negatives) are directly impacted by the proportion of positive and negative instances in the dataset. As shown in Table 1 and Table 2, the XGB method achieved MCC values of 84.70% and 83.62%, respectively, clearly surpassing the values obtained by other methods.

In the same vein, when analyzing the kappa index, XGB reached nearly 85% for the final variable of mortality, marking a significant advancement over KNN and DT by 5.4% and 8.2%, respectively. As for the final variable of hospital stay, XGB approached about 84%. This trend persists when considering the AUC and DYI parameters, where XGB surpasses the rest of the methods with higher values. These findings emphasize the superior ability of XGB to accurately categorize COVID-19 patients treated with remdesivir in terms of mortality and/or hospital stay.

**Figure 2:**
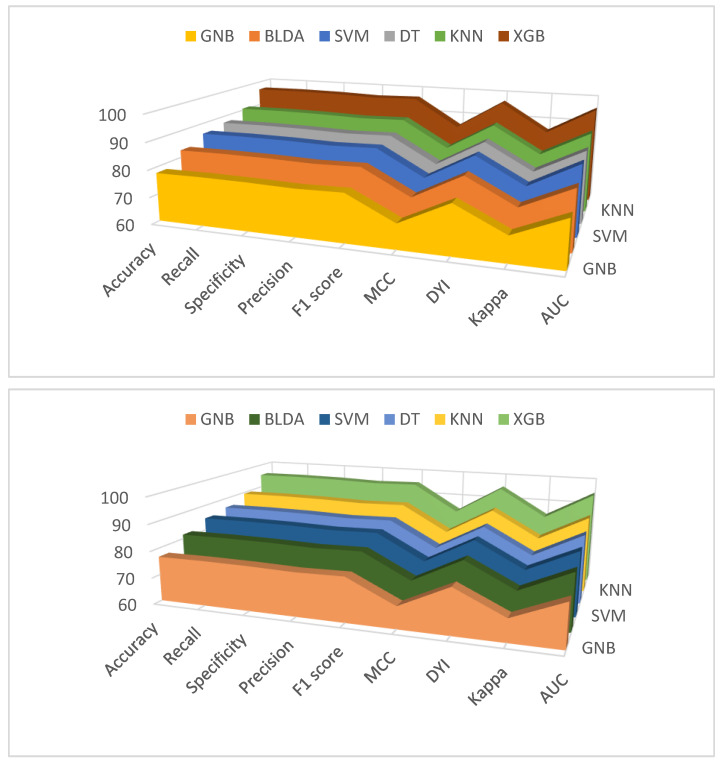
Graphic representation of different metrics for each of the machine learning models used, in percentage. Training phase (**above**) and test phase (**below**) for predicting mortality in COVID-19 patients undergoing remdesivir treatment. Abbreviations: AUC: area under curve; BLDA: Bayesian linear discriminant analysis; DT: decision tree; DYI: degenerated Younden index; GNB: Gaussian naïve Bayes; KNN: K-nearest neighbor; MCC: Matthew’s correlation coefficient; SVM: support vector machine; XGB: extreme gradient boost.

**Figure 3:**
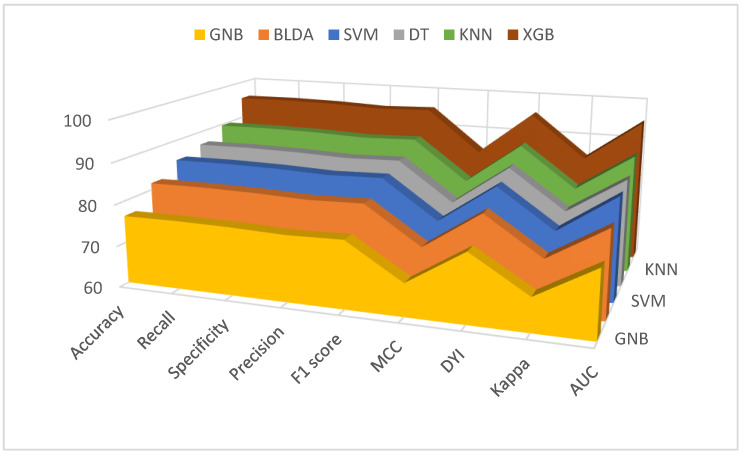

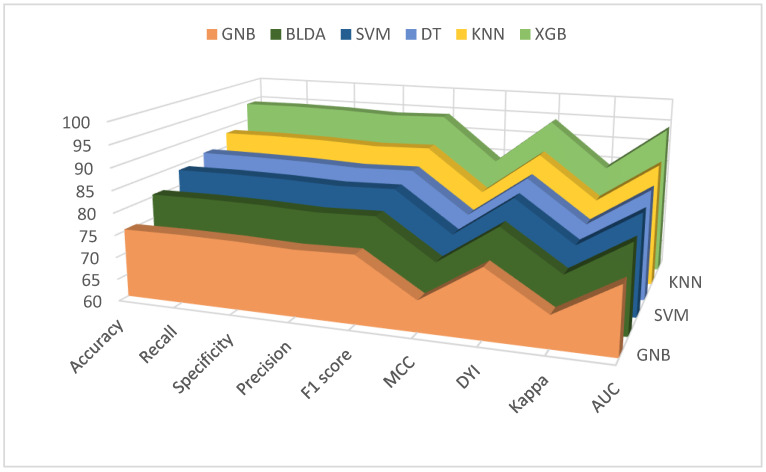
Graphic representation of different metrics for each of the machine learning models used, in percentage. Training phase (**above**) and test phase (**below**) for predicting hospital stay in COVID-19 patients undergoing remdesivir treatment. Abbreviations: AUC: area under curve; BLDA: Bayesian linear discriminant analysis; DT: decision tree; DYI: degenerated Younden index; GNB: Gaussian naïve Bayes; KNN: K-nearest neighbor; MCC: Matthew’s correlation coefficient; SVM: support vector machine; XGB: extreme gradient boost.

Figure 2 and Figure 3 provide a comprehensive analysis, comparing the performance of the XGB method with alternative classifiers across a variety of essential metrics, for both mortality and hospital stay, respectively. These metrics include balanced accuracy, recall, specificity, precision, *F*_1_ score, CKI, MCC, AUC, and DYI.

**Figure 4:**
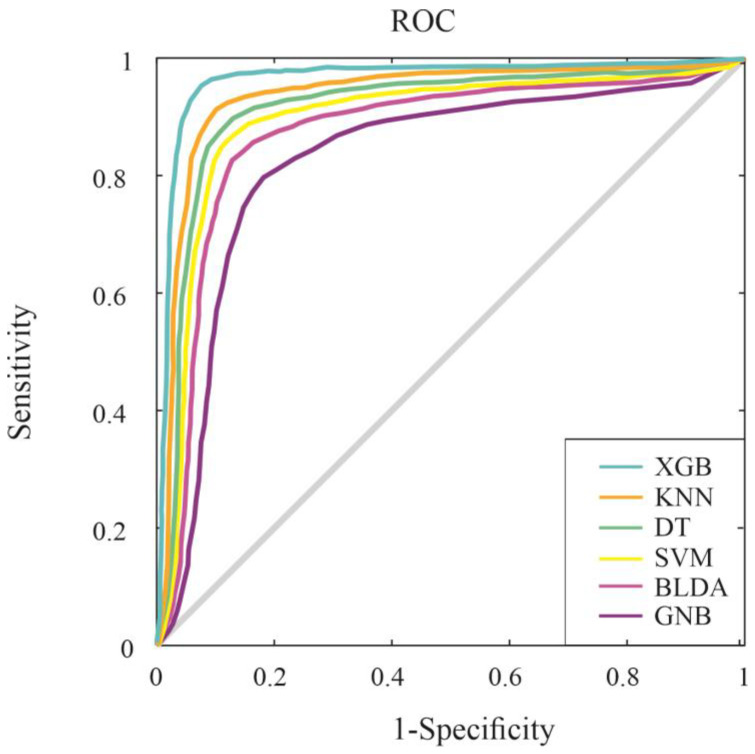

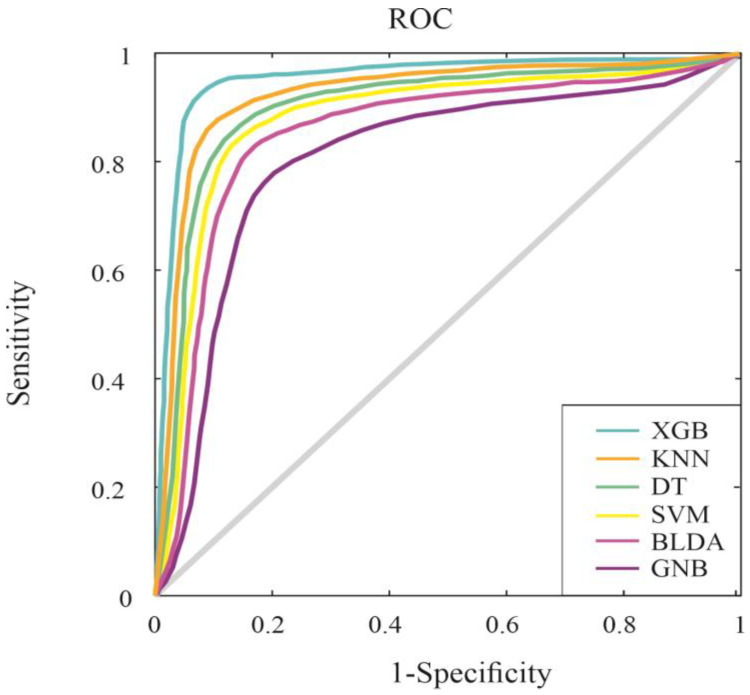
ROC curves for mortality (**above**) and hospital stay (**below**) for the six assessed machine learning predictors. Abbreviations: BLDA: Bayesian linear discriminant analysis; DT: decision tree; GNB: Gaussian naïve Bayes; KNN: K-nearest neighbor; ROC: receiver operating characteristic; SVM: support vector machine; XGB: extreme gradient boost.

Furthermore, the ROC curve is a crucial tool used for assessing and comparing how effectively the proposed system classifies compared to other machine learning methods. It is constructed by plotting sensitivity against specificity across various threshold values. Figure 4 depicts the outcomes of different classification systems, aligning with the primary goal of categorizing patients in the study.

In particular, the XGB method exhibits a significantly larger area under the ROC curve, indicating its enhanced ability to accurately classify the two distinct classes, both in terms of mortality and hospital stay. This is further supported by the specific numerical values provided in Table 1 and Table 2.

**Figure 5:**
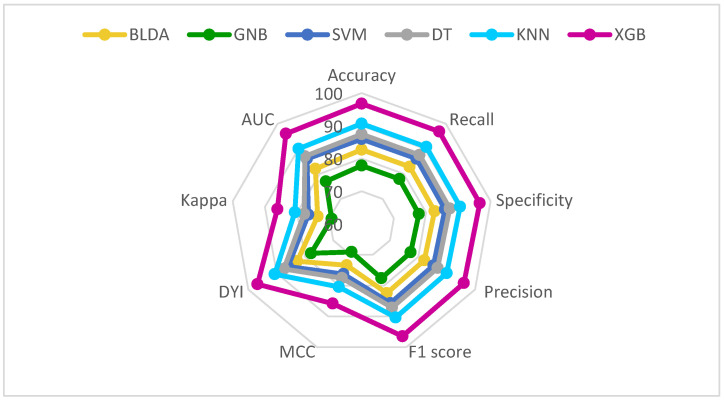

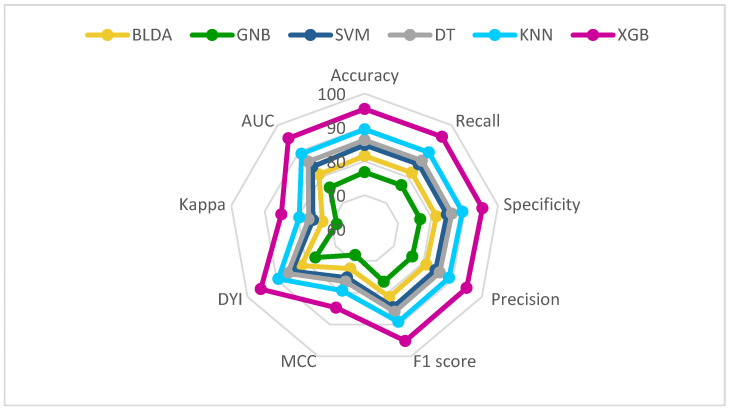
Radar plot depicting the training phase (**above**) and test phase (**below**) for predicting mortality in COVID-19 patients undergoing remdesivir treatment. AUC: area under curve; BLDA: Bayesian linear discriminant analysis; DT: decision tree; DYI: degenerated Younden index; GNB: Gaussian naïve Bayes; KNN: K-nearest neighbor; MCC: Matthew’s correlation coefficient; SVM: support vector machine; XGB: extreme gradient boost.

**Figure 6:**
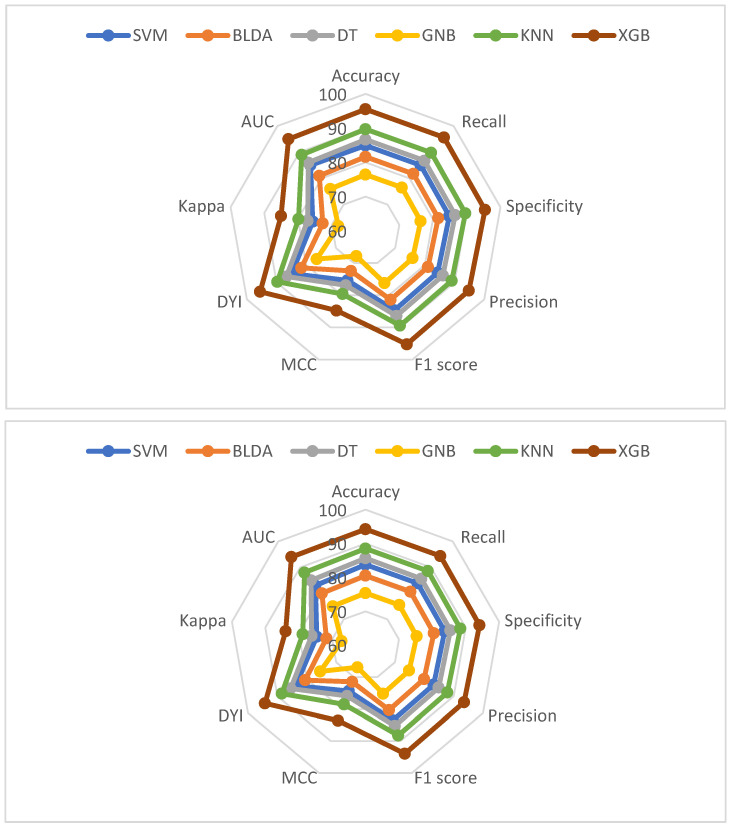
Radar plot depicting the training phase (**above**) and test phase (**below**) for predicting hospital stay in COVID-19 patients undergoing remdesivir treatment. AUC: area under curve; BLDA: Bayesian linear discriminant analysis; DT: decision tree; DYI: degenerated Younden index; GNB: Gaussian naïve Bayes; KNN: K-nearest neighbor; MCC: Matthew’s correlation coefficient; SVM: support vector machine; XGB: extreme gradient boost.

For better clarity, we have arranged all metrics for each dataset—both training and validation—and represented them in a radar plot (Figure 5 and Figure 6). In an ideal situation where the model performs exceptionally well in all metrics, the plot would create a circle covering the entire grid. In our study, the training sets consistently show higher scores across all metrics, whereas the validation sets generally display lower scores.

The radar plots give us a quick look at the model’s performance. A bigger circle on the validation set suggests a better predictive method. Figure 5 shows that our suggested XGB system is a good example of a well-balanced model. Importantly, both the training and validation sets have similar radar plots, indicating neither overfitting nor underfitting. This boosts the model’s ability to work well with new inputs, delivering accurate results effectively.

On the flip side, the GNB method consistently comes in as the least effective performer in all metrics. Based on these findings, we can confidently state that our proposed XGB system excels at categorizing patients in line with the study’s goals. It provides high accuracy and automation, making it a valuable tool for clinical practice.

**Figure 7:**
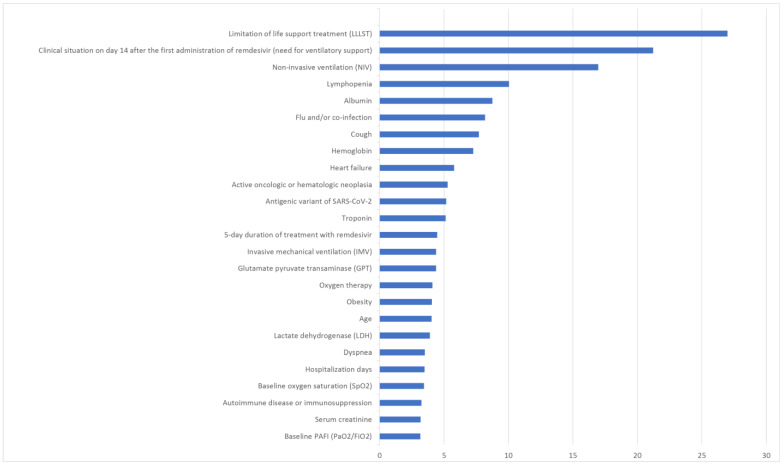

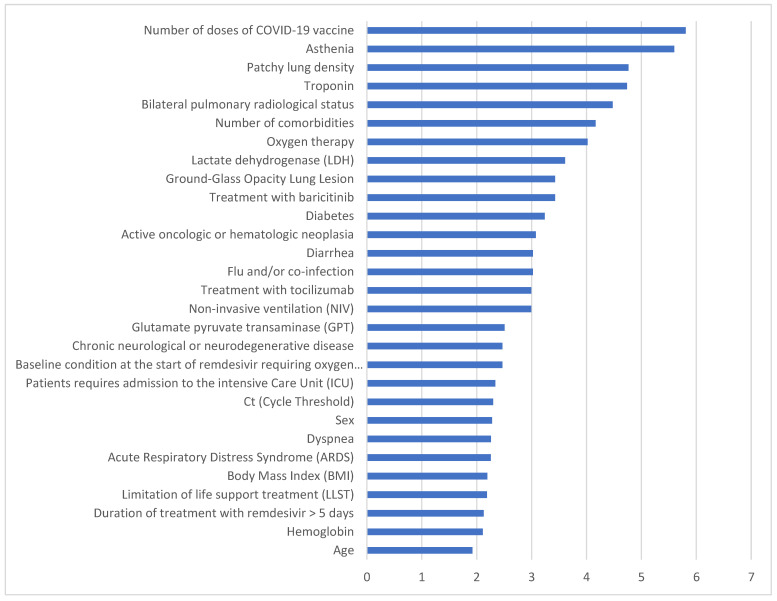
Graphical representation of the predictive variables with the most significant impact on classifying COVID-19 patients undergoing remdesivir treatment in terms of mortality (**above**) and hospital stay (**below**).

With the proposed XGB method, the predictive factors linked to a poorer outcome in COVID-19 patients treated with remdesivir, in terms of mortality, include limitation of life support treatment, a need for ventilatory support (especially IMV) on day 14 after the first dose of remdesivir, lymphopenia, low levels of albumin and hemoglobin, the presence of flu and/or coinfection, and cough. Factors associated with a worse outcome of remdesivir use in terms of hospital stay include the number of doses of the COVID-19 vaccine, patchy lung density, bilateral pulmonary radiological status, the number of comorbidities, oxygen therapy, troponin and LDH levels, and asthenia. Figure 7 shows a bar graph displaying the weights of predictive variables that notably improve the classification accuracy of different ML methods.

The main baseline clinical data of the 252 patients included in the study are presented in Table 3.

## 4. Discussion

Among the approved medications for COVID-19, remdesivir stands out as the preferred antiviral treatment for hospitalized patients infected with SARS-CoV-2. Additionally, other drugs like tocilizumab and baricitinib show promise, particularly for severe cases, including those requiring IMV [32]. While remdesivir can reduce viral levels and shorten symptom duration, identifying the patients who benefit most from it remains uncertain. This knowledge is crucial for minimizing unnecessary side effects and costs while optimizing resource use [33].

The European Medicines Agency (EMA) initially granted conditional approval for marketing across the EU on 3 July 2020, which later transitioned to full marketing authorization on 8 August 2022. This medication is approved for use in adults and children as young as 4 weeks old, weighing at least 3 kg, who have pneumonia and need supplemental oxygen (either low- or high-flow oxygen or other non-invasive ventilation at the start of treatment). Additionally, it can be used in adults and children weighing at least 40 kg who do not need supplemental oxygen but are at higher risk of developing severe COVID-19 [34].

On the other hand, AI has been employed to identify genomic sequences of SARS-CoV-2, including antigenic variants, as well as to develop drugs and vaccines for COVID-19 [35]. It has also been used to discover drug combinations against COVID-19 [36].

Drug repurposing trials have aimed at finding potential treatments for COVID-19, including antiviral therapies, anti-inflammatory drugs, antithrombotic agents, and immunomodulators [37]. In a study by Basit SA et al., a deep learning model was employed to predict the effectiveness of different medications, identifying remdesivir as highly effective against COVID-19 with a 95% positive score [38].

Understanding the physiopathology of COVID-19 can help scientists develop effective antiviral drugs by uncovering unknown viral pathways and structures. With the advancements in AI and ML, it is reasonable to use these methods to explore new candidates. Various studies, like the one by Imtiaz F and Pasha MK, have focused on examining the structure of the RNA-dependent RNA polymerase (RdRp) using ML techniques. RdRp is crucial for virus replication and holds potential as a promising target for COVID-19 treatment [39]. Remdesivir, which transforms into an analog of adenosine triphosphate during intracellular metabolism, works mainly by integrating into the developing RNA chain by the RdRp. This disrupts viral replication, a key aspect of its antiviral activity [12]. Monitoring the impact of emerging mutations on viral replication and response to antiviral drugs is essential. Remdesivir shows stability as an RdRp inhibitor compared to other antivirals in the presence of mutations at this level of viral replication [40].

The WHO’s Solidarity trial was the first major study to show the limited clinical effectiveness, in terms of mortality, of three repurposed antivirals in hospitalized COVID-19 patients: lopinavir, hydroxychloroquine, and interferon (IFN)-β1a. The remdesivir arm continued, with 4146 patients receiving remdesivir and 4129 assigned to the control group. It was observed that remdesivir does not have a significant effect on COVID-19 patients already on ventilation. Among other hospitalized patients, it has a minor impact on mortality or progression to ventilation [17]. Remdesivir may be beneficial in the clinical course for both hospitalized and non-hospitalized patients, but certainty remains limited [41]. There is evidence supporting the clinical benefit of a 5-day regimen of remdesivir in patients with moderate COVID-19 infection (lung infiltrates and SpO_2_ in ambient air > 94%) [14]. For patients with severe COVID-19 (SpO_2_ ≤ 94% while breathing ambient air and radiological evidence of pneumonia) who do not require mechanical ventilation, there does not seem to be a significant difference between a 5-day and a 10-day course of remdesivir [15]. Remdesivir significantly reduces hospitalization days and lowers inflammatory markers compared to standard treatment in patients with moderate to severe COVID-19 [42]. Compared to standard care, remdesivir quickly improves low oxygen levels (reducing the need for ventilatory support) and reduces inflammation (lowering IL-6 levels), leading to a better course of moderate to severe COVID-19 [43]. Patients treated with remdesivir spend less time in the ICU and have better survival rates [44].

Recent meta-analyses confirm that the use of remdesivir can help reduce mortality in COVID-19 patients and shorten the time to clinical improvement [32,45]. Observational studies have demonstrated benefits in hospital mortality with remdesivir therapy [46,47].

Remdesivir appears to lower mortality rates in hospitalized COVID-19 patients who do not require oxygen support or only need standard oxygen therapy. However, it does not seem to help patients on mechanical ventilation [48]. A recent meta-analysis by Huang C et al. found that hospitalized adult COVID-19 patients who did not need extra oxygen or only required low-flow oxygen and were treated with remdesivir had a lower risk of death. However, those needing high-flow oxygen or IMV did not see the same benefit [49]. Remdesivir also seems to speed up recovery, reduce complications, and might slightly decrease the need for ventilation [32,50,51]. Additionally, a 5-day treatment course appears to provide more benefits with fewer side effects and lower costs for non-ventilated patients compared to a 10-day course [50].

Remdesivir is safe to use, but when combined with corticosteroids, it does not seem to offer extra clinical benefits [45]. However, when paired with baricitinib, it is not only safe but also seems to be more effective than using remdesivir alone. This combination can reduce recovery time and speed up clinical improvement in COVID-19 patients, particularly those needing high-flow oxygen or NIV [52].

This study leads the way in developing, comparing, and evaluating six supervised ML methods to predict factors that reduce the effectiveness of remdesivir in hospitalized patients with SARS-CoV-2 pneumonia. We collected data on 133 demographic, clinical, and laboratory variables. Among the ML algorithms tested, XGB stood out as the best performer, achieving the highest balanced accuracy rates for predicting mortality (95.4%) and hospital stay duration (94.2%).

When SARS-CoV-2 infects cells in the respiratory tract, it causes damage and triggers the immune system to release proinflammatory substances like IFNγ, IL-1β, IL-6, and TNF-α [53]. Among these, IL-6 is particularly crucial, as it escalates inflammation from mild to severe states, such as cytokine release syndrome (CRS) and ARDS. These conditions can be fatal for severely ill COVID-19 patients, with mortality rates surpassing 70% [8,54].

In our investigation, we observed a hospital mortality rate of 13.5%, with 3.9% of all patients requiring IMV. These findings align with those of other studies [17,48,55]. The median duration of hospitalization following the administration of remdesivir was 8 days (Interquartile Range, IQR, 5–12).

Our analysis involved testing various ML classifiers, among which the XGB method stood out as the most precise in identifying patients at higher risk of mortality and/or prolonged hospital stay. After a thorough examination, we compared the XGB model with several other supervised ML methods commonly found in the existing literature, such as BLDA, GNB, DT, KNN, and SVM. It is important to note that in biomedical scenarios, current ML classification techniques consistently outperform unsupervised methods, achieving higher average accuracy rates for both regression and classification tasks [56]. In our study, BLDA and GNB performed the poorest among the methods examined, while KNN’s performance closely matched that of XGB. These findings align with previous research on the predictive capabilities of supervised ML algorithms for COVID-19 mortality and hospitalization durations [57,58].

In our study, we used a radar graph to assess the performance of ML models in both the training and testing phases. The results indicate that the XGB model performs exceptionally well, especially in managing large datasets without overfitting. It surpasses other methods by achieving superior precision, recall, and overall accuracy. The reliable performance of the XGB model makes it incredibly valuable, especially in biomedical applications like predicting cancer stages for patients [59].

In our cohort, 58.3% were male, with a median age of 77 (IQR 66.7–85.2). We found several factors linked to negative outcomes after receiving remdesivir, notably regarding mortality and hospital stay. For mortality, adverse outcomes were associated with life support limitations, the need for ventilatory support (particularly IMV) 14 days after the initial remdesivir dose, lymphopenia, low albumin and hemoglobin levels, flu and/or coinfection, and cough. When it comes to hospital stay, factors associated with a worse outcome with remdesivir use included the COVID-19 vaccine doses, patchy lung density, bilateral pulmonary radiological status, comorbidity count, need for oxygen therapy, high levels of troponin and LDH, and the presence of asthenia.

These identified factors provide valuable insights into the potential determinants of adverse outcomes associated with remdesivir use. In the realm of research on factors influencing the response to remdesivir in hospitalized patients with COVID-19, few studies have been conducted [60,61,62].

Previous studies have identified factors that predict the severity and mortality of COVID-19 patients, but these were conducted before standard treatment with remdesivir was introduced. ML techniques have confirmed that demographic factors (like age); clinical factors (such as comorbidities or symptoms); and analytical factors are associated with the severity, mortality, and length of hospital stay of COVID-19 patients, regardless of the specific treatment used [63,64]. Adamidi et al.’s systematic review also found predictors of disease progression and mortality using ML techniques, similar to our study. They emphasized that age, PCR and LDH levels, lymphopenia, and chest X-ray and CT scan findings are commonly linked to adverse outcomes in COVID-19 patients [58]. Additionally, other studies have highlighted the effectiveness of the XGB method in predicting adverse outcomes in hospitalized COVID-19 patients [65].

In Choi YJ et al.’s study, multivariate analysis confirmed that a high National Early Warning Score (NEWS) and Charlson Comorbidity Index (CCI) at admission, along with dyspnea, were independent risk factors for 30-day mortality in COVID-19 pneumonia patients treated with remdesivir and dexamethasone [60]. NEWS is based on a scoring system that combines six key physiological measurements (respiratory rate, SpO2, temperature, systolic blood pressure, heart rate, and level of consciousness) upon hospital admission or monitoring. This tool is highly sensitive and specific in predicting early mortality in prehospital and emergency department settings [66]. It emphasizes that COVID-19 admissions carry a significantly higher mortality risk compared to non-COVID-19 admissions, highlighting the elevated baseline mortality risk associated with COVID-19 [67].

In our study, factors indicating a higher risk of mortality, similar to NEWS and shortness of breath, included the limitation of life support treatment and the need for ventilatory support, especially IMV, 14 days after the initial dose of remdesivir. Regarding hospital stay, a similar factor was the requirement for oxygen therapy. These respiratory parameters and symptoms indicate respiratory failure and suggest a more severe decline in hospitalized COVID-19 patients.

Several studies confirm that the main clinical symptoms in patients admitted with COVID-19 are shortness of breath, cough, and fever [66]. In our study, cough was associated with a higher risk of mortality, while asthenia was linked to prolonged hospital stay.

In the study conducted by Choi YJ et al., similar to our findings, the majority of hospitalized cases of COVID-19 pneumonia occurred in adults aged ≥65 years; thus, there were no significant differences in the prognosis based on age. Additionally, within the laboratory parameters, low lymphocyte count, high levels of CRP, and elevated LDH indicated an unfavorable prognosis [60]. In our study, we found that lymphopenia, along with low levels of albumin and hemoglobin, was strongly linked to mortality in patients treated with remdesivir. Lymphopenia indicates an impaired T-cell response and weakened adaptive immunity. SARS-CoV-2 infection mainly impacts T lymphocytes, particularly CD4+ and CD8+ T cells, resulting in decreased counts. Lymphopenia is more commonly seen in severe cases [68]. Georgakopoulou VE et al. confirmed that low levels of albumin and the C-reactive protein to albumin ratio were predictors of mortality, similar to our findings [62]. For hospital stays, elevated troponin and LDH levels were the most relevant predictors, along with observing bilateral lung involvement and patchy lung density in imaging tests. In the study by Terkes V et al., advanced age, elevated CRP, and the Computed Tomography (CT) score were identified as significant predictors of disease outcome [69]. However, the intense inflammatory response triggered by the infection can lead to alterations in hemostasis and coagulation parameters [70]. In our research, platelet count was not a significant predictor to influence the sought-after final outcome.

On the other hand, comorbidities themselves result from inflammation and can induce a proinflammatory state. The CCI is a simple, easy-to-apply, and valid method for classifying comorbidities and estimating mortality from COVID-19 [71].

Recent studies have taken into account the pre-existing health conditions (comorbidities) of patients infected with SARS-CoV-2 and their association with the progression of the disease in terms of mortality and hospital stay [61].

Aging and pre-existing health conditions can create a state of meta-inflammation, amplifying inflammation in COVID-19 and increasing the risk of mortality. Several studies demonstrate a correlation in patients affected by COVID-19 between age, number of comorbidities, and certain laboratory markers [72]. There appears to be a positive link between inflammation biomarkers such as CRP, ferritin, and LDH and the number of comorbidities in COVID-19 patients. The same pattern is observed in hematological parameters like the neutrophil-to-lymphocyte ratio. Similar to these studies, in our research, the number of comorbidities was associated with worse outcomes for patients and extended hospital stays.

Chronic kidney disease, the incidence of acute kidney injury, and atrial fibrillation have been shown to be comorbidities associated with reduced survival in patients hospitalized for COVID-19 [73]. Other studies confirm that lymphopenia, often observed in cancer patients, is associated with a higher risk of mortality [74]. Hematological disorders are also seen as additional health conditions that can negatively impact the effectiveness of antiviral treatments such as remdesivir [75]. In other studies, hypertension and type 2 diabetes, linked with obesity as metabolic syndrome, are considered significant risk factors for adverse outcomes [76]. In our study, the presence of influenza and/or co-infection constituted the comorbidity associated with higher mortality.

In the recent meta-analysis by Amstutz A et al., neither age, comorbidities, nor the use of corticosteroids had an impact on the effectiveness of remdesivir in terms of mortality [48]. Similarly, the need for increased respiratory support in patients has shown limited effectiveness of remdesivir in reducing mortality, aligning with our own research outcomes [17].

In our investigation, 22.2% of patients developed ARDS according to the latest definition [77]. ARDS is a clinical syndrome of acute hypoxemic respiratory failure due to lung inflammation, not caused by cardiogenic pulmonary edema. Various studies using these techniques have found that ARDS is associated with fatal outcomes in COVID-19 patients, making ventilatory support essential, including IMV [58,64]. In our study, both IMV and oxygen therapy were predictors of poor prognosis.

In contrast to Choi YJ et al.’s study, in ours, a lower number of COVID-19 vaccine doses was linked to clinical deterioration and a longer hospital stay [60]. The study by Georgakopoulou VE et al. confirmed that, regardless of vaccination status, pre-existing comorbidities, age, and gender, patients with a combination of biomarkers indicating acute inflammatory response, cell death, and hypercoagulability—specifically, CRP, LDH, and fibrinogen—reflected the severity of COVID-19 [78]. More recently, Mikulska M et al. demonstrated that receiving fewer doses of the COVID-19 vaccine was a predictive factor for treatment failure with antivirals such as remdesivir [75]. Several studies consistently show that individuals who are not vaccinated or receive fewer vaccine doses are more likely to experience negative outcomes, including the need for mechanical ventilation or death during hospitalization [79]. This aligns with our findings.

In the study by Shimizu H et al., the time intervals between symptom onset, diagnosis, and antiviral treatment were significant predictors of moderate illness [80]. In our study, following the applied protocol, remdesivir treatment was supposed to commence upon microbiological confirmation of SARS-CoV-2 infection and within 7 days of clinical symptom onset.

None of the drug treatments, including early antibiotic use, had a significant effect on the final outcome, similar to findings in other studies [60].

Most current studies using ML techniques confirm that respiratory parameters like SpO_2_ and the need for invasive ventilatory support are considered the most important predictors for mortality in hospitalized COVID-19 patients treated with remdesivir. While hypertension and worsening renal function are also considered mortality predictors in these studies, they did not hold enough significance in our research [81,82]. Kuno T et al. developed a predictive model for in-hospital mortality using ML methods in COVID-19 patients treated with steroids and remdesivir. Variables associated with mortality included age, hypertension, SpO_2_, blood urea nitrogen, ICU admission, and endotracheal intubation [81].

On the other hand, evidence indicates the presence of different COVID-19 patient phenotypes showing diverse inflammatory and immune responses, mortality risks, and treatment outcomes [83,84]. In a study by Chen H et al., two COVID-19 phenotypes emerged: hypo-inflammatory and hyper-inflammatory, with the latter marked by heightened pro-inflammatory cytokine levels and increased complication rates. Corticosteroid therapy was linked to lower 28-day mortality (HR, 0.45; 95% CI, 0.25–0.80; *p* = 0.0062) in the hyper-inflammatory type [84]. The lack of efficacy with remdesivir treatment may lend support to this idea, underscoring the importance of identifying factors for personalized treatments.

Our study has both limitations and strengths. The main limitations come from its retrospective, single-center design and limited sample size. Yet, these limitations are offset by leveraging robust methodological tools such as ML. ML methods have the advantage of being effective even with small datasets, resulting in simple and fast classification for our proposed method. We have also used data-augmentation techniques to enhance our analysis [30].

One strength of our study is its inclusion of a diverse patient population, covering individuals with common health conditions often overlooked in standard clinical trials. This broader representation allows our findings to be applicable to a wider range of patients. Our methodology effectively identifies patients who could benefit from remdesivir, potentially leading to better survival rates and shorter hospital stays. Additionally, similar research using ML techniques has identified factors linked to worse outcomes in severe COVID-19 patients treated with tocilizumab [85]. Comparative studies suggest that ML methods may offer greater accuracy and efficiency compared to traditional logistic regression analysis, particularly with limited sample sizes.

The XGB method is a straightforward binary classification system that is user-friendly and easy to train. As more data are collected, this algorithm improves its prediction accuracy.

## 5. Conclusions

Remdesivir has proven to be beneficial in patients with SARS-CoV-2 pneumonia, especially in those without critical illness criteria. However, a significant number of patients still die or require longer hospital stays despite treatment with remdesivir. Therefore, we utilized ML techniques, which are increasingly important in predicting important events. Out of the six supervised ML methods we tested, XGB demonstrated the highest accuracy in predicting factors linked to poorer outcomes, such as mortality and hospital stay length, in hospitalized COVID-19 patients treated with remdesivir. This tool can help healthcare professionals make timely and impactful clinical decisions to optimize remdesivir treatment for COVID-19 patients who meet specific clinical criteria.

## Figures and Tables

**Table 1:** Summary of the mean values and standard deviation of balanced accuracy, recall, precision, *F*_1_ score, AUC, MCC, DYI, and Kappa index of the machine learning models and the novel method proposed in this study for determining mortality.

Methods	Balanced Accuracy	Recall	Precision	*F*_1_ Score
SVM	84.80 ± 0.75	84.90 ± 0.73	84.20 ± 0.74	84.55 ± 0.73
BLDA	81.61 ± 0.84	81.70 ± 0.81	81.03 ± 0.82	81.36 ± 0.81
DT	86.25 ± 0.71	86.35 ± 0.70	85.63 ± 0.71	85.99 ± 0.70
GNB	76.80 ± 0.94	76.89 ± 0.93	76.25 ± 0.95	76.57 ± 0.94
KNN	89.43 ± 0.60	89.54 ± 0.58	88.79 ± 0.59	89.16 ± 0.58
XGB	95.45 ± 0.46	95.56 ± 0.45	94.77 ± 0.47	95.17 ± 0.44
**Methods**	**AUC**	**MCC**	**DYI**	**Kappa**
SVM	0.84 ± 0.02	75.25 ± 0.75	84.80 ± 0.73	75.49 ± 0.74
BLDA	0.81 ± 0.03	72.41 ± 0.82	81.61 ± 0.81	72.65 ± 0.81
DT	0.86 ± 0.02	76.53 ± 0.71	86.25 ± 0.71	76.78 ± 0.72
GNB	0.76 ± 0.03	68.15 ± 0.95	76.80 ± 0.94	68.37 ± 0.94
KNN	0.89 ± 0.02	79.35 ± 0.57	89.43 ± 0.58	79.62 ± 0.58
XGB	0.95 ± 0.02	84.70 ± 0.42	95.45 ± 0.46	84.98 ± 0.43

Abbreviations: AUC: area under curve; BLDA: Bayesian linear discriminant analysis; DT: decision tree; DYI: degenerated Younden index; GNB: Gaussian naïve Bayes; KNN: K-nearest neighbor; MCC: Matthew’s correlation coefficient; SVM: support vector machine; XGB: extreme gradient boost.

**Table 2:** Summary of the mean values and standard deviation of balanced accuracy, recall, precision, *F*_1_ score, AUC, MCC, DYI, and Kappa index of the machine learning models and the novel method proposed in this study for the hospital stay.

Methods	Balanced Accuracy	Recall	Precision	*F*_1_ Score
SVM	83.80 ± 0.77	83.89 ± 0.76	83.20 ± 0.78	83.54 ± 0.76
BLDA	80.54 ± 0.85	80.64 ± 0.84	79.94 ± 0.86	80.30 ± 0.85
DT	85.67 ± 0.73	85.61 ± 0.72	84.90 ± 0.75	85.25 ± 0.74
GNB	75.41 ± 0.98	75.50 ± 0.95	74.86 ± 0.96	75.18 ± 0.97
KNN	88.53 ± 0.66	88.61 ± 0.64	87.85 ± 0.67	88.24 ± 0.68
XGB	94.24 ± 0.48	94.35 ± 0.44	93.57 ± 0.46	93.96 ± 0.47
**Methods**	**AUC**	**MCC**	**DYI**	**Kappa**
SVM	0.83 ± 0.02	74.35 ± 0.74	83.80 ± 0.77	74.60 ± 0.73
BLDA	0.80 ± 0.03	71.47 ± 0.83	80.54 ± 0.85	71.71 ± 0.84
DT	0.85 ± 0.03	75.89 ± 0.71	85.51 ± 0.73	76.13 ± 0.71
GNB	0.75 ± 0.03	66.91 ± 0.92	75.43 ± 0.95	67.15 ± 0.93
KNN	0.88 ± 0.02	78.53 ± 0.63	88.51 ± 0.66	78.79 ± 0.64
XGB	0.94 ± 0.02	83.62 ± 0.41	94.24 ± 0.45	83.90 ± 0.42

Abbreviations: AUC: area under curve; BLDA: Bayesian linear discriminant analysis; DT: decision tree; DYI: degenerated Younden index; GNB: Gaussian naïve Bayes; KNN: K-nearest neighbor; MCC: Matthew’s correlation coefficient; SVM: support vector machine; XGB: extreme gradient boost.

**Table 3:** Main basal clinical data of patients. Data are *n* (%) or median (IQR), unless otherwise stated.

Variable	Cohort
Number of patients	252
Age (years) (IQR)	77 (66.7–85.2)
Male, *n* (yes %)	147 (58.3)
Hospital admission (days) after remdesivir administration (IQR)	8 (5–12)
Exitus, *n* (yes %)	34 (13.5)
Patients with duration of remdesivir treatment for 4–5 days, *n* (yes %)	194 (76.9)
Patients with duration of remdesivir treatment > 5 days, *n* (yes %)	14 (5.6)
Duration (days) from onset of symptoms to microbiological confirmation (IQR)	2 (1–4)
Oxygen therapy, *n* (yes %)	188 (74.6)
Patients who need admission to the ICU, *n* (yes %)	14 (5.6)
Limitation of life support treatment, *n* (yes %)	47 (18.6)
IMV, *n* (yes %)	10 (3.9)
Baseline situation at the start of remdesivir: - Patient does not require supplementary oxygen, *n* (yes %) - Patient requires low-flow oxygen therapy, *n* (yes %)	
	96 (38.1)
	156 (61.9)
Hypertension, *n* (yes %)	158 (62.7)
Diabetes, *n* (yes %)	89 (35.3)
Dyslipemia, *n* (yes %)	112 (44.4)
Smoker, *n* (yes %)	55 (21.8)
Obesity, *n* (yes %)	45 (17.8)
COPD, *n* (yes %)	35 (13.9)
Heart failure, *n* (yes %)	51 (20.2)
Ischemic heart disease, n (yes %)	46 (18.2)
Chronic kidney disease, n (yes %)	26 (10.3)
Chronic neurological or neurodegenerative disease, *n* (yes %)	62 (24.6)
Mental health disorder, *n* (yes %)	60 (23.8)
Active hematological or oncological neoplasia, *n* (yes %)	63 (25.0)
Patients with ≥3 comorbidities, *n* (yes %)	193 (76.6)
Fever, *n* (yes %)	148 (58.7)
Cough, *n* (yes %)	156 (61.9)
Dyspnea, *n* (yes %)	158 (62.7)
Asthenia, *n* (yes %)	106 (42.1)
Presence of flu and/or coinfection, *n* (yes %)	29 (11.5)
Angiotensin-converting enzyme inhibitors/angiotensin receptor blockers, *n* (yes %)	109 (43.2)
Antibiotics, *n* (yes %)	234 (92.8)
Immunosuppressants and/or immunomodulators, *n* (yes %) - Corticoides, *n* (yes %) - Tocilizumab, *n* (yes %) - Baricitinib, *n* (yes %)	197 (78.2)
	124 (49.2)
	33 (13.1)
	32 (12.7)
Albumin (g/dL) (IQR)	3.4 (2.9–3.6)
Hemoglobin (g/dL) (IQR)	12.4 (10.9–13.7)
Troponin (ng/mL) (IQR)	12 (7–27)
CRP (mg/dL) (IQR)	6.9 (2.4–14.1)
LDH (U/L) (IQR)	463 (359.5–609.5)
Ferritin (µg/L) (IQR)	413 (193–782)
D-dimer (ng/mL) (IQR)	878 (499.7–1534.2)
Creatinina (mg/dL) (IQR)	0.8 (0.6–1.1)
CK (U/L) (IQR)	98.5 (51.0–192.0)
PAFI (IQR)	323.8 (257.0–368.0)
Lymphocytes (10^9^/L) (IQR)	0.8 (0.6–1.2)
Bilateral lung radiological status, *n* (yes %)	98 (38.9)
Ground-glass opacity lung injury, *n* (yes %)	69 (27.4)
Patchy lung density, *n* (yes %)	64 (25.4)
Patients with ≥3 doses of COVID-19 vaccine, *n* (yes %)	199 (78.9)
Acute Respiratory Distress Syndrome, *n* (yes %)	56 (22.2)
Clinical status on day 14 after the first administration of remdesivir - Hospitalized and requires supplementary oxygen but not IMV, *n* (yes %) - Hospitalized and requires IMV, *n* (yes %)	
	23 (9.1)
	5 (1.9)

Abbreviations: CK: creatine kinase, COPD: chronic obstructive pulmonary disease, CRP: C-reactive protein, ICU: intensive care unit, IQR: interquartile range, IMV: invasive mechanical ventilation, LDH: lactate dehydrogenase, PAFI: ratio of arterial oxygen partial pressure (PaO_2_) to fractional inspired oxygen (FiO_2_).

## Data Availability

The datasets used and analyzed in this study can be obtained by contacting the corresponding author upon request.
